# New type of SSUrDNA sequence was detected from both *Plasmodium ovale curtisi* and *Plasmodium ovale wallikeri* samples

**DOI:** 10.1186/1475-2875-13-216

**Published:** 2014-06-03

**Authors:** Mei Li, Zhigui Xia, He Yan

**Affiliations:** 1 National Institute of Parasitic Diseases, Chinese Centre for Diseases Control and Prevention, Key Laboratory of Parasite and Vector Biology, Ministry of Public Health, WHO Collaborating Centre for Malaria, Schistosomiasis and Filariasis, Shanghai 200025, People’s Republic of China

## Abstract

**Background:**

*Plasmodium ovale* is relatively unfamiliar to Chinese staff engaged in malaria diagnosis. In 2013, dried blood spots of four unidentified but suspected ovale malaria samples were sent to the National Malaria Reference Laboratory (NMRL) for reconfirmation.

**Methods:**

Partial and complete, small, subunit ribosomal DNA (SSU rDNA) sequences of four samples were obtained with PCR-cloning-sequencing method. Obtained sequences were analyzed by aligning with each other and with nine SSU rDNA sequences of six known *Plasmodium* parasites. A phylogenetic tree was constructed based on complete SSU rDNA sequences and 12 same gene sequences derived from six known *Plasmodium* parasites and three *Babesia* parasites. Primary structure of conservative and variable regions of variant sequences was determined also by comparing them with those of six known *Plasmodium* parasites. To confirm their existence in genome, they were redetected with primers matching their variable regions. PCR systems aimed to roughly detect any eukaryotes and prokaryotes respectively were also applied to search for other pathogens in one of four patients.

**Results:**

Totally, 19 partial and 23 complete SSU rDNA sequences obtained from four samples. Except eight variant sequences, similarities among sequences from same DNA sample were in general high (more than 98%). The phylogenetic analysis revealed that three cases were infected by *P. ovale wallikeri* and one by *P. ovale curtisi*. Four of the variant sequences which obtained from four samples relatively showed high similarities with each other (98.5%-100%). Identical variant sequences actually could be re-obtained from each DNA sample. Their primary structure of conservative and variable regions showed quite fit with that of six known *Plasmodium* parasites. The test for prokaryote pathogens showed negative and the tests for eukaryotes only found DNA sequences of Human and *P. ovale* parasites.

**Conclusion:**

Both *P. ovale wallikeri* and *P. ovale curtisi* infections are present in imported malaria cases of China. New type of partial SSU rDNA sequence which assumed to express in a certain life stage of *P. ovale* was obtained from both *P. ovale wallikeri* and *P. ovale curtisi* samples. This discovery would supply information and clues to identify and understand *P. ovale* parasites more accurately.

## Background

*Plasmodium ovale* was the last described malaria parasites of humans [1]. However, relatively little attention had been paid to it since that time, because of its commonness, mild symptoms, usually low parasitaemia and same treatment method with *P. vivax*[2]. With the application of molecular methods in malaria diagnosis, more understanding of it had been achieved. Based on DNA polymorphisms in small subunit ribosomal RNA (SSU rRNA) gene, *P. ovale* parasites were defined into two types, a classic type and a variant type [3-8]. Lately, they were further confirmed based on the characterization of cysteine protease gene, ookinete surface protein gene and cytochrome b gene. Recently, these two types were certified to occur globally and so were formally named *Plasmodium ovale curtisi* (typical type) and *Plasmodium ovale wallikeri* (variant type), respectively in honour of Christopher F Curtis (1993–2008) and David Walliker (1940–2007) [9].

Ovale malaria was commonly found in tropical West Africa and infrequently reported in Asia and Oceania [2,8-17]. In China, ovale malaria transmission and local infection was seldom reported except some occasionally imported cases. In a series of articles introduced the malaria situation in China during 2000–2012 [18-29], the ovale malaria situation was mentioned only since 2011 when the first 4 Provincial Reference Laboratories (PRLs) for malaria were established. Since that, 17 and 41 imported ovale malaria cases were confirmed respectively in laboratories in 2011 and 2012 [28,29]. So, *P. ovale* was relatively unfamiliar to Chinese staff engaged in malaria diagnosis, and *P. ovale* subspecies were even stranger for they are seldom differentiated in Chinese laboratories.

According to the requirement of China’s National Malaria Elimination Programme (NMEP), every malaria case must be confirmed by the polymerase chain reaction (PCR) method. The nested-PCR system produced by Snounou in 1993 (NP-1993) [30] was recommended and widely adopted in PRLs. With the development of the reference system in China, staff began to be familiar with *P. ovale*. Unfortunately, microscopy positive but nested-PCR negative cases began to arise. In early 2013, dry blood spots of four unidentified but suspected ovale malaria patients were sent to the national malaria reference laboratory for reconfirmation.

Besides, stage-specific SSU rRNAs transcribed from structurally distinct nuclear genes had also been reported in some *Plasmodium* parasites, such as *Plasmodium berghei*, *Plasmodium vivax* and *Plasmodium falciparum*[31-35]. One type of these SSU rRNA genes is expressed in the asexual bloodstream and hepatic stage (A gene), and the other type is expressed in the mature parasites (sporozoites) of the mosquito (C gene or named S gene in *P. vivax*) [31-33]. The third gene was O gene which was sampled and cloned from *P. vivax* and expressed in the maturing oocysts [34]. However, different stage-specific SSU rRNAs genes in *P. ovale* and *Plasmodium malariae* had not been reported.

## Methods

### Ethical clearance

The study was reviewed and approved by the Ethical Committee of National Institute of Parasite Diseases (NIPD), China Centre of Disease Control (CDC), and written informed consent was obtained from all the participants. No additional blood samples were taken from patients, other than those required for the primary diagnosis of malaria.

### Sample information and DNA sample preparation

Dried blood spots of four malaria patients infected in Africa were supplied by Dalian CDC (labeled DL), Guizhou CDC (labeled GZ), Hainan CDC (labeled HN) and Shanghai CDC (labeled SH). The DL patient who was diagnosed in early 2012 had been to Equatorial Guinea. GZ, HN and SH patients who were diagnosed in late 2012 or early 2013 had been to Sierra Leone and Angola respectively. Genome DNA of them was prepared using QIAamp DNA Mini Kit (Quigen, 51306).

### Cloning and sequencing of SSU rDNA sequences of all samples

All the samples were first reviewed with nest-PCR systems of NP-1993 [30]. The PCR products of the first round (partial SSU rDNA sequences) were cloned into pMDTM19-T Vector (Takara Vector Cloning Kit, 6013) and propagated in *Escherichia coli* DH5 cells under ampicillin selection. Positive plasmids were purified using Generay plasmid mini kit (Shanghai Generay Biotech Co Ltd, China, GK2004-100) and sequenced with Double DNA chain termination method (Shanghai MAP Biotech Co Ltd) on 3730XL DNA Analyzer. The plasmid DNA was assembled with software of DNA sequence assembly.

To confirm the results deduced from partial SSU rDNA sequences, primers to amplify complete SSU rDNA sequences were designed with software Oligo 6.0 based on complete SSU rDNA sequences of *P. ovale wallikeri* (Accession No. AB182491) download from National Center for Biotechnology Information (http://www.ncbi.nlm.nih.gov/)*.* The amplified primers were UF: 5′-AACCTGGTTGATCTTGCCAGTAGTC-3′ and RL: 5′-TAATGATCCTTCCGCAGGTTC ACC-3′. The PCR, cloning and sequencing processes were the same as that used in obtained partial SSU rDNA sequences*.*

### Sequences analysis

Assembled sequences amplified with same primers were aligned in software DNAman6.0. Conflicts among sequences were resolved by majority vote. Voted sequences were analyzed by aligning them with each other and with nine SSU rDNA sequences of six known *Plasmodium* parasites, *P. falciparum* (A and S type), *P. vivax* (A, S and O type), *P. malariae*, *Plasmodium knowlesi*, *P. ovale curtisi*, *P. ovale wallikeri*. GenBank accession numbers of nine SSU rDNA sequences were: M19172, M19173, U03079, U03080, U93235, M54897, AM910985, AB182491, AB182489. A phylogenetic tree was constructed based on complete SSU rDNA sequences and 12 same gene sequences from six known species and three species of *Babesia* parasites, *Babesia rodhaini*, *Babesia.microti* and *Babesia divergens* with software Mega 5.0, respectively. GenBank accession numbers of *Babesia* parasites were M87565, AB190459, AY046576.

The characteristics of variant SSU rDNA sequences were determined by comparing them with nine SSU rDNA sequences of known *Plasmodium* parasites with the help of software DNAman 6.0. The classification standard to determine the conservative or variable regions were quoted from Qari, 1994 [32]. Regions at which all sequences showed about 90% identity with each other were classified as conservative regions, < 60% as variable regions, and 60-90% as moderate similarity regions.

To confirm their presence in *P. ovale* genome, new primers, which matched the variable areas of variant sequence, were designed with software Oligo 6.0. They were shown in Table 1.

**Table 1:** Specific primers detecting variant SSU rDNA sequences in 4 DNA samples

**Reaction turn**	**Primer sequences**	**Nucleotide position**	**Product size**
First round			
PoWu	5′-CGTTTCTGAGGTGCTACGCTTGG-3′	R2 (Low similarity)	910 bp
PoWL1	5′-GACGACACTCGACTCGGTTATCC-3′	R7(Low similarity)	
Second round			
PoWu	5′-CGTTTCTGAGGTGCTACGCTTGG-3′	R2(Low similarity)	440 bp
PoWL2	5′-GTATCTGATCGTCTTCACTCCCTT-3′	R4(High similarity)	

### Searching for other pathogens in HN patient

Primers conserved for eukaryotes: NS1, 5′-GTAGTCATATGCTTGTCTC-3′, NS2, 5′-GGCTGCTGGCACCAGACTTGC-3′ and for prokaryotes: 27F, 5′- AGA GTT TGA TCM TGG CTC AG-3′, (M = C or A) and 1429R, 5′- TAC GGY TAC CTT GTT ACG ACT T-3′,(Y = C or T) were selected to search for other pathogens in HN patient. The PCR products which presented DNA bands on the gel was recovered and cloned into plasmid and sequenced.

## Results

### Molecular diagnosing results with nest-PCR

**Figure 1:**
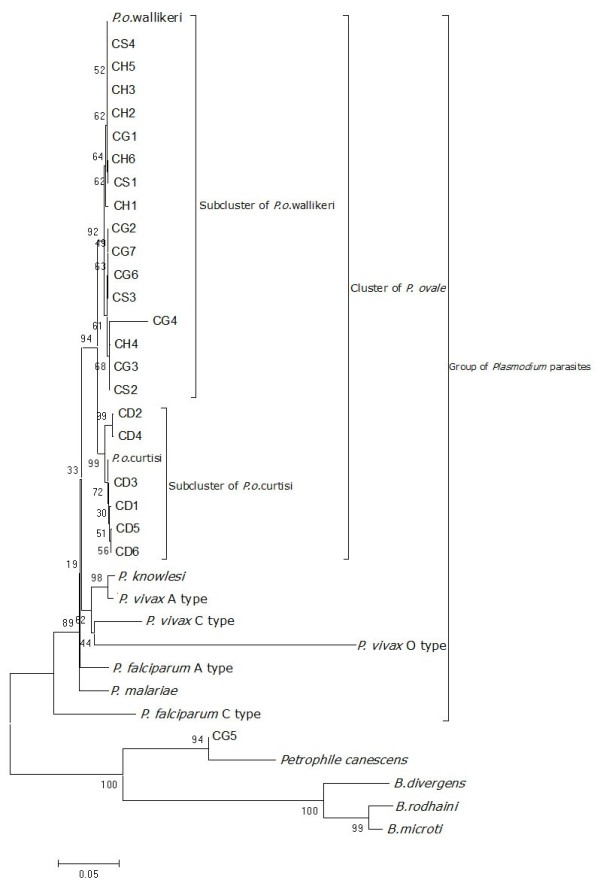
**Phylogenetic tree representing the relationship of complete SSU rDNA sequences from four cases to another 13 taxa.** cDL1-cDL6: 4 complete SSU rDNA sequences from Dailian sample, Genbank accession No.s were KF696369-KF696375; cHN1-cHN6: 6 complete SSU rDNA sequences from Hainnan sample, Genbank accession No.s were KF018654-KF018659; cGZ1-cGZ7: 7 complete SSU rDNA sequences from Guizhou sample, Genbank accession No.s were KF696359-KF696365; cSH1-cSH4: 4 complete SSU rDNA sequences from Shanghai sample, Genbank accession No.s were KF219558-KF219561.

With NP-1993 system [30], except positive control samples of *P. falciparum* (205 bp) and *P. vivax* (120 bp), only DL sample showed *P. ovale* positive (800 bp) while the other three showed negative results (Figure 1). Therefore, DL patient should be infected by *P. ovale*.

### Characteristics of partial SSU rDNA sequences

Nineteen sequences were obtained from four DNA samples. Eleven of them showed closest relationship with *P. ovale wallikeri* or *P. ovale curtisi* with high identities (more than 98%). Eight of them showed the closest relationship with *P. ovale wallikeri* but with relatively low identities (79%-92%).

Four assembled sequences (numbered from pDL1-pDL4) of 1,069 bp-1,087 bp belonging to the DL sample were rigorously elected from 12 positive clones. Identities among them varied from 75.8 to 99.6%. Sequences of pDL1, pDL2 and pDL3 showed closest relationship with *P. ovale curtisi* with identities of more than 98%. But pDL4 showed the closest relationship to *P. ovale wallikeri* with identity of 79%.

Three assembled sequences (numbered from pGZ1-pGZ3) of 1,085 bp-1,090 bp belonging to GZ sample were rigorously elected from 20 positive clones. Identities among them varied from 89.1 to 95.9%. All of them showed closest relationship with *P. ovale wallikeri* with relatively low identities of 86%-90%.

Seven assembled sequences (numbered from pHN1-pHN7) of 1,061 bp-1,091 bp belonging to the HN sample were rigorously elected from 23 positive clones obtained from four batches of reaction products. Identities among them varied from 79.2 to 99.4%. Among them, the identities (91%, 85% and 92%) of sequences pHN1, pHN3 and pHN5 with that of *P. ovale wallikeri* were much lower than others (more than 98%).

Five assembled sequences (numbered from pSH1-pSH5) of 1,062 bp-1,091 bp belonging to the SH sample were rigorously elected from ten positive clones of SH sample. Identities among them varied from 74.3 to 99.8%. But pSH3 showed an identity of 85% with SSU rDNA sequence of *P. ovale wallikeri* while the other sequences showed more than 98.5%.

### Characteristics of complete SSU rDNA sequences

In total, 23 complete SSU rDNA sequences were obtained from four DNA samples. They all showed closest relationship with *P. ovale wallikeri* or *P. ovale curtisi* with high identities (more than 98%) but low identities (74.4%-90.3%) with eight variant sequences obtained in this study. For DL sample, six different assembled sequences (2,104 bp) were obtained from ten positive clones (cDL1-cDL6). The identities among themselves were 99.0-99.8%. Except of pDL4, identities between pDL and cDL sequences were between 98.3% and 99.7%. No complete SSU rDNA sequences had identities with pDL4 were more than 78.2%.

For GZ sample, seven different assembled sequences (1,915 bp-2,094 bp) of complete SSU rRNA gene were obtained from 18 positive clones and numbered cGZ1-cGZ7. The identities among themselves were between 69.7-99.8%. Most variations presented in sequences of CG4 and CG5 which only showed about 90 and 81% identities with other sequences. There were rich variations among obtained cGZ sequences and pGZ sequences (with identities of 70.3%-90.3%).

For the HN sample, six different assembled sequences (2,091 bp-2,096 bp) were obtained from ten positive clones (CH1-CH6). The identities among themselves varied between 98.55-99.9%. Except of pHN1, pHN3 and pHN5, identities between all pHN sequences and cHN sequences were between 98.0%-99.4%. No complete SSU rDNA sequences whose identities with pHN1, pHN3 and pHN5 were more than 90%.

For SH sample, four assembled sequences (2,092 bp-2,094 bp) of complete SSU rRNA gene were obtained from eight positive clones (cSH1-cSH4). The identities among them were between 98.4 to 99.6%. Except of pSH3, the identities between all pSH sequences and cSH sequences were more than 98.1% (98.1%- 99.7%). The identity of PS3 with complete SSU rDNA sequences was between 74.4 and 75.0%.

### Phylogenetic analysis of complete SSU rDNA sequences

The bootstrap consensus neighbouring-joining (NJ) tree (Figure 1) showed that most of the complete SSU rDNA sequences obtained in this study gathered in the cluster of *P. ovale*. Among them, most of the sequences from HN, GZ and SH samples gathered in the subcluster in which *P. ovale wallikeri* was included, with bootstrap of 92%. But no distinctively phylogenetic relationship presented between SSU rDNA sequences from three different samples. These results strongly supported all three cases being infections with P*. ovale wallikeri*. All the sequences obtained from DL sample made up the *P. ovale* cluster with *P. ovale curtisi* (bootstrap =99%). The sequence cGZ5 and the shrub stood out of *Plasmodium* parasites but together with *Babesia* parasites. The phyogenetic results were concordant with the reference deduced from sequences aligning results that HN, GZ and SH showed closest relationship with *P. ovale wallikeri*, but DL with *P. ovale curtisi*.

### Redetection of new variant SSU rDNA sequences

With the new nested PCR system, DNA fragments of aimed size of 440bp were present on the gel picture of all PCR products of four DNA samples. And their sequencing results of the PCR products showed that they were identical with pGZ1, PS3, PH3 and pDL4 respectively. However, no DNA bands present on the gel picture of the first round PCR products. This indicated that variant sequences truly existed in *P. ovale*. However, their copies in genome might be not as much as shown in positive clones.

### Characteristics of variant SSU rDNA sequences

Summarily, among eight variant sequences, pGZ1 and pHN1 were identical with each other and showed 99.9% identities with pSH3. These three sequences were all derived from *P. ovale wallikeri* samples and showed 97.9% identities with pDL4, which derived from *P. ovale curtisi* sample. Besides, two sequences from different samples, pGZ2 and pHN5 were showed high similarity of 99.4% with each other too. Similarities between variant sequences and known *P. ovale* SSU rDNA sequences were all less than 92% as described previously. Same situation also presented between different *Plasmodium* species and different SSU rDNA types of same species.

The frequency of amplification and cloning of each the 8 variant SSU rDNA sequences among all sequences obtained from that isolate was calculated and is shown in Table 2. The four similar variant SSU rDNA sequences, pDL4, pGZ1, pHN3, and pSH3, showed relatively high percentage than other variant sequences (33-90%). Among four of them, pDL4 showed the lowest amplified rate, which was 33%, and pGZ1 showed the highest applied rate, which was 90%. Each of the other four variant sequences, pGZ2, pGZ3, pHN1, and pHN5 only presented once.

**Table 2:** The occurred percentage of eight variant SSU rDNA sequences among the positive sequence clones

**Name of the sequences**	**pDL4**	**pGZ1**	**pGZ2 + pGZ3**	**pHN3**	**pHN1 + pHN5**	**pSH3**
Percentage in positive clones	33% (4/12)	90% (18/20)	10% (2/20)	35% (8/23)	9% (2/23)	50% (5/10)

Based on the alignment results, the primary structure of variant sequences was determined according to varying similarity of different regions between 10 DNA sequences of *P. ovale* (Additional file 1). They were divided into eight regions (R1-R8), four high similarity regions, two low similarity regions and two moderate similarity regions (Additional file 1 and Table 3). At R1, R4, R6 and R8, all variant sequences were showed high similarity with each other and those of *P. ovale wallikeri* (PoW) and *P. ovale curtisi* (PoC) (93%-100%). So these regions were high similarity or conservative regions. At R2 and R7, similarity of 39%-54% presented among most of the aligned sequences sourced from *P. ovale.* So, they were low similarity or variable regions. At R3 and R5, the similarity among them arranged within 62%-81%. So they were moderate similarity regions.

**Table 3:** **Comparison of primary structure of conservative and variable regions between variant sequences and other *Plasmodium *** parasites*^1^

**Block no**		**Nucleotide position**		**Similarity**		**(%)**		
PvC	pGZ1	PvC	pGZ1	vPvC	pGZ1	PvC	pGZ1-1*^3^	pGZ1-2*^4^
B5	R1	54-656	1-44*^2^	High	High	95	100	100
B6	R2	657-804	45-207	Low	Low	26	39-40	36
B7	R3	805-927	208-333	Moderate	Moderate	74	78-81	76
B8	R4	928-1,112	334-517	High	High	92	93	93
B9	R5	1,113-1,194	575-603	Low	Low-Moderate	49	62-65	52
B10	R6	1,195-1,468	605-876	High	High	97	97	97
B11	R7	1,467-1,687	878-1,033	Low	Low	34	53-54	40
B12	R8	1,688-1,793	1,034-1,090	High	High	94	93-95	95

*^1^Data of the other *Plasmodium* parasites and classification standard were quoted from Qari [32].

*^2^One nucleotide base (A) on sequence of *P. vivax* C type (PVC) (Nucleotide position 656) deleted on PG1.

*^3^Similarity between PG1 and corresponding sequences of *P. ovale wallikeri* and *P. ovale curtisi*.

*^4^Similarity between PG1 and corresponding sequences of *P. vivax* C type.

Comparing the primary structure of variant sequences with that of nine known SSU rDNA sequences of *Plasmodium* species indicated that the division of R1-R8 could completely fit with that of Block5 – Block12 of complete *Plasmodium* SSU rRNA sequences displayed by Qari in 1994 (Additional file 1 and Table 3). Though similarities between DNA sequences of *P.ovale* were little more than those between different known *Plasmodium* species, the arrangement and positions of conservative, variable and moderate similarity regions of them were completely same.

Beside of the same structure of variant sequences with known *Plasmodium* parasites, the other interesting point was found. As shown in Additional file 1, the similarities of variant sequences with PoW or PoC varied greatly at different variable regions. Sequences pDL4, pGZ1, pHN3 and pSH3 showed high variable from PoW or PoC at all of four variable regions. But the other 4 sequences, pGZ2, pGZ3, pHN1 and pHN5 showed high variable from Pow only in some variable regions. Specifically, pHN1 showed nearly identical with PoW at R2 and R3 regions, pGZ2 at R7, pGZ3 at R5 and R7, and pHN5 at R7. At the other regions, they showed nearly identical with pGZ1 but high variations from Pow (Table 4).

**Table 4:** Detail distributions of 4 variable regions in 8 variant SSU rDNA sequences

	**R2**	**R3**	**R5**	**R7**
pDL4	X	X	X	X
pGZ1	X	X	X	X
pGZ2	X	X	X	/
pGZ3	X	X	/	/
pHN1	/	/	X	X
pHN3	X	X	X	X
pHN5	X	X	X	/
pSH3	X	X	X	X

Additionally, these four variable regions had selected as the targeted sites, designing primers to distinguish different *Plasmodium* species. For example, the R2 and R7 were the targeted sites of the ovale species-specific primers of rOVA1 and rOVA2 matched to [30].

### No other pathogen found in HN patient

The PCR results with prokaryote primers showed negative while that with eukaryote primers showed positive. Sequencing results of six positive clones recovered from the main band turned out to be SSU rDNA of *P. ovale* parasites and the other six from the smears belonged to human DNA sequences.

## Discussion

Malaria diagnosis was one of key steps of malaria control or elimination. Usually, three methods, that is microscopy, RDT and PCR, are used to detect malaria parasites. Unfortunately, the low and decreasing morbidity of malaria cases brought huge challenges to microscopists. Thus, clinicians or public health workers relied on RDT or PCR in malaria diagnosis more and more. However, quite a few RDT products were insensitive to some *P. ovale*. Besides, *P. ovale* often co-infected patients with *P. falciparum* or other *Plasmodium* parasites [5,10-12,16,36] and sometimes with a very low parasitaemia. So, if the PCR method could not find them out in time too, ovale patients might be delayed or neglect treatment. Then ovale malaria transmission or relapse might be resulted from the parasites lingered in patients’ livers [15,37,38] which would threat the populations seriously.

SSU rRNA gene was the most widely accepted molecular marker in identifying different malaria parasites infecting humans with PCR method. The common PCR systems based on SSU rDNA sequences were NP-1993 [30]. Latterly, NP-2002 [39] and NP-2005 [36] were developed as new variant SSU rDNA sequences were discovered. At first, the *P. ovale wallikeri* made the NP-1993 system unreliable [7,36] and the system of NP-2002 seemed to resolve this problem, in which not only the PCR product size of first round extended from 1,100 bp to 1,700 bp along the forward direction of SSU rDNA sequences, but the reverse ovale-specific primer was designed at conserved region. However, another PCR system of NP-2005 in which a pair of new *P. ovale* species-specific primers was applied, proved to be more accurate than both NP-1993 and NP-2002 in detecting *P. ovale*. For the present, the problem is that one of the species-specific primers used in these three systems is located at the variable region R2 or B6. In this region, variant sequences obtained in this study showed low similarity (<60%) with normal ones. But high similarity (>90%) presented between variant and normal ones where genus-specific primers usually matched. So, theoretically, all the SSU rDNA types could be amplified synchronously with same genus-specific primers. If most of the PCR products obtained in the first round reaction did not match the species-specific primer (as shown in this study), all of the PCR results in the three systems might show negative. So, new system which could detect both *P. ovale curtisi* and *P. ovale wallikeri* was required. However, the glad thing for hospitals or CDCs which owned real-time quantitative PCR instruments was that some new assays developed in recent years might not be borrowed to apply. For example, in the assay created by Bauffe in 2012 [13], the forward primer POF, 5′-ATAAACTATGCCGACTAGGTT-3′ matching to *P. ovale curtisi* and *P. ovale wallikeri* specifically covered both R4 and R5 and possessed 2 nts different from variant sequences, while the reverse primer POR, 5′-ACTTTGATTTCTCATAAGGTACT-3′ covered both R5 and R6 and possessed 1 nt different from variant sequences. So the variant sequences could not bother it.

For the variant sequences, the possibility had been suspected that they might derive from the other pathogen which co-infected patients with *P. ovale*. But two excuses denied it. The first was that no other *Plasmodium* parasites were found with specific primers to complete SSU rDNA sequences and no other pathogens were detected with PCR systems specific to both prokaryote and eukaryote organisms. The second was that these four patients were infected by *P. ovale* parasites in different time and at different places, and furthermore they also lived faraway from each other in China. So the other assume was suggested that they might belong to one type of SSU rRNA gene which expressed in a certain life stage of *P. ovale* parasites as A, C and O types of SSU rRNA genes did in *P. falciparum* and *P. vivax*. As known, three SSU rRNA gene types were confirmed in *P. vivax*, two in *P. falciparum* and three in *P. berghei*[31-35]. However, only one type of SSU rDNA sequences was identified in each of the *P. ovale* species. Except of their same primary organization of conservative and variable regions, the little higher similarities (99.9%-100%) among variant sequences (pGZ1, pHN3 and pSH3), which derived from *P. ovale wallikeri* patients than that (97.9%) between variant sequences derived from different *P. ovale subspecies* supported this assumption too. Otherwise, analogous sequences were found by other scientists. So it was confident to believe that the variant sequences belong to one type of SSU rDNA sequence of *P. ovale*. Whatever, true evidence such as analysis on transcript of *P. ovale* sporozoites or genome DNA of *P. ovale* is required to confirm this assume.

In the result of this study, there was a conflict in that no complete SSU rDNA sequences showed high similarity with the variant sequences. There may be two reasons: either the primers were not conserved enough or there were fewer copies of variant SSU rDNA than the normal ones. For the first excuse, the primers used to amplify complete SSU rDNA sequences were conserved in all the other five *Plasmodium* parasites, so the conflict unlikely resulted from it. Therefore, it seems that the different copies of them in the whole genome DNA had led to the results. Regrettably, no such research has mentioned it.

Except of pDL4, pGZ1, pHN3 and pSH3, there were other 4 special variant sequences, pGZ2, pGZ3, pHN1 and pHN5 were also detected. Their character showed high similarities with PoW in some variable regions and low similarities in the other variable regions. This result indicated that hybridization or recombination might have occurred between different types of SSU rRNA genes. But more studies were required to discover if they had really happened in the process of ovale parasites development or just were missing PCR products.

In a word, variant sequences obtained from *P. ovale* samples on the one hand might make some PCR systems invalid, but on the other hand, they supply information and clues to identify and understand *P. ovale* parasites more accurately.

## Competing interests

The authors declare that they have no competing interests.

## Authors’ contributions

ZGX carried out the revising manuscript draft and collected the samples and their epidemic information of four patients. HY carried out the DNA preparation and PCR diagnosis with NP-1993 method. ML finished the other work. All authors read and approved the final manuscript.

## Supplementary Material

Additional file 1 **Aligning results of variant sequences with SSU rDNA sequences of six known *Plasmodium *** **parasites and their primary structure of conservative and variable regions.** Dots represent similarity with *P. ovale wallikeri* (PoW) and dashes represent gaps introduced to align the sequences. PvA, PvO and PvS represent A, O and S SSU rDNA sequences of *P. vivax*; PfA and PfS represent A and S of *P. falciparum*; Pm, Pk and PoC represent *P. malariae*, *P. knowlesi* and *P. ovale curtisi*. Click here for file
