# Supplementary material for: New type of SSUrDNA sequence was detected from both Plasmodium ovale curtisi and Plasmodium ovale wallikeri samples
Source: Malar J. 2014 Jun 3;13:216. doi: 10.1186/1475-2875-13-216 (PMC4049480; doi:10.1186/1475-2875-13-216)
Supplement: Additional file 1 — Aligning results of variant sequences with SSU rDNA sequences of six known Plasmodium parasites and their primary structure of conservative and variable regions. Dots represent similarity with P. ovale wallikeri (PoW) and dashes represent gaps introduced to align the sequences. PvA, PvO and PvS represent A, O and S SSU rDNA sequences of P. vivax; PfA and PfS represent A and S of P. falciparum; Pm, Pk and PoC represent P. malariae, P. knowlesi and P. ovale curtisi. [file 1475-2875-13-216-S1.docx]

R1

R2

PoW TTAAAATTGTTGCAGTTAAAACGCTCGTAGTTGAATTTCAAAGAATCA----ATATTTTAAGTAATGC--------------TT---TTGGTATAAGATGCTTAGG----CAATACAAC------GTATCTGCTCTTTGC 140

PoC ................................................----..............A.--------------..---...C..............A----.........------.......T....... 140

pSH3 ............................................G...TGGCG.T.C.G.G..GC.A.GCT-TGGTTG---A.C-GCCCA.C.CTGACCATC..T.ATTTT.T.GT.GA------AC.GTGAGC..GG.T 140

pDL4 ............................................G...TGGCG.T.C.G.G..GC.C.--T-TGGTCG---A.CTGCCCA.C.CTGAC--TC..T.ATTTT.T.GT.GA------.C.GTGTGA..GG.T 140

pGZ1 ............................................G...TGGCG.T.C.G.G..GC.A.GCT-TGGTTG---A.C-GCCCA.C.CTGACCATC..T.ATTTT.T.GT.GA------AC.GTGAGC..GG.T 140

pGZ2 ............................................G...TGGCG.T.C.G.G..GC.A.GCT-TGGTTG---A.C-GCCCA.C.CTGACCATC..T.ATTTT.T.GT.GA------AC.GTGAGC..GG.T 140

pGZ3 ............................................G...TGGCG.T.C.G.G..GC.A.GCT-TGGTTG---A.C-GCCCA.C.CTGACCATC..T.ATTTT.T.GT.GA------AC.GTGAGC..GG.T 140

pHN1 ................................................----................--------------..---...................----.........------............... 140

pHN3 ............................................G...TGGCG.T.C.G.G..GC.A.GCT-TGGTTG---A.C-GCCCA.C.CTGACCATC..T.ATTTT.T.GT.GA------AC.GTGAGC..GG.T 140

pHN5 ............................................G...TGGCG.T.C.G.G..GC.A.GCT-TGGTTG---A.C-GCCCA.C.CTGACCATC..T.ATTTT.T.GT.GA------AC.GTGAGC..GG.T 140

PvSvS .............................................C.----A.........-...C..CGT-TAGCTA--GA.CCACAA..GG.TGAGCCAA.CACGGTTTCG-G.TT----------------..G... 140

PvA ...............................................----G..........C..C..TTC-TAGCTT--AA.CCACA.AAC-.G.T.CT--.C---GTATCG-..T-----------------T.G... 140

PvO .......C..........T..T.T........A.....G........----A.C........C..C..GTT-CAGCTT--AA.CCACAC.AC-.GGTGCT--.C---GTATCG-GTTGGTACTTA.C...GA.AT.G... 140

PfA ...............................................----G........TTGT.ACTATT-CTAGGG--GAAC------TA-.TTT.GCT..T---GGCTTT--------------------AA.AC.. 140

PfS .......................T............A.T........C---G..G...C.TT...ACTGGT-TTGGGA--AAACCAAA.ATA-.T.T..AT..T---GCTTTG-TTC.A--------------AA.AA.G 140

Pm .........................................G.....----A................TTTGTATATTTATAACAAAG.T...C.TT.A.AA..AACGCCA.GCGTT.TATTTTTTCTGT.A.AT....T 140

Pk ...............................................----G.............C..TTC-TAGCTACAAA.CCACA.AAC-.G.TGCC--.CC.CGTATCG-..T-----------------T.G... 140

R3

R2

PoW ATTCCTTATCCAA-AATGTGTTCTTATTATAAAAAGGATTCTTTTTAAAATCTCCTT---------TACTTTTT---------GTACTGGAGATTTTGTTACTTTGAGTAAATTAGAGTGTTCAAAGCAAACAGTTAAA- 280

PoC .........G...-........................................T..---------.G..A...TTTA-----...T....................................................- 280

pSH3 G..TAGCTC.A.G-..A..TG...CC..TAC...GT....T..C.A.GGG....T.GAG-------...CAG.C---------C.TGG.AG.T.C..T....C...................T...........A..C.- 280

pDL4 G...GA.T..A.G-..A..-G...CC..TAC...GT....T..C.A.GGG....T.GAG-------...CAG.C---------C.TGG.AG.T.C..T....C...................T...........A..G.- 280

pGZ1 G..TAGCTC.A.G-..A..TG...CC..TAC...GT....T..C.A.GGG....T.GAG-------...CAG.C---------C.TGG.AG.T.C..T....C...................T...........A..C.- 280

pGZ2 G..TAGCTC.A.G-..A..TG...CC..TAC...GT....T..C.A.GGG....T.GAG-------...CAG.C---------C.TGG.AG.T.C..T....C...................T...........A..C.- 280

pGZ3 G..TAGCTC.A.G-..A..TG...CC..TAC...GT....T..C.A.GGG....T.GAG-------...CAG.C---------C.TGG.AG.T.C..T....C...................T...........A..C.- 280

pHN1 .............-...........................................---------........---------........................................................- 280

pHN3 G..TAGCTC.A.G-..A..TG...CC..TAC...GT....T..C.A.GGG....T.GAG-------...CAG.C---------C.TGG.AG.T.C..T....C...................T...........A..C.- 280

pHN5 G..TAGCTC.A.G-..A..TG...CC..TAC...GT....T..C.A.GGG....T.GAG-------...CAG.C---------C.TGG.AG.T.C..T....C...................T...........A..C.- 280

PvS GCAT.C..C.T.T-C.A.C...T..T.A..T...GT.T...............T...---------...C..AACCATA-------TG.A.........................A........T.........A..C.- 280

PvA GCATT..-G.T.T-T..........T.A..T....T............GGA..TTC.---------.TGC..CGGCTT---------G.A..TCC.......................................A..T.- 280

PvO GCATT..-G.T.C-T.C........C.A..T....T...........GGGGT.T.C.---------.TG...CGGCAT---------..A.TCCC.......C...C.A...A..A.C.........G.........T.T 280

PfA T.C.TC...TA-T-T....TC.T--TAA....C..A..........-...ATC..CACTTT-----.G.....GCT-------T.TT...G................................................- 280

PfSfS T..T..A..AA.T-T....T..TA.CAG...TG.CA..A.......T...ATCT...CAATA----.G.....ATTGC-----T.TTGA...G...................A.TA........T.A..G..G.G..GTC 280

Pm T..AT.A..AT.TAT...C................T...............TCTT..GTGTAA---.TT...A.GCAT----------..GA...............................................- 280

Pk GCATT...G.T.T-T..........T.A..T....T............G..TCATC.ATTAAGAAT.TGC..CGGCATAATTTT.TT..AT...C.......................................A..T.- 280

R4

R3

PoW -----GCATTTTACTGCGTTT-GAATACTACAGCATGGAATAACAAAATTGAACAAGTCAAA-ACTCT---GTTCTTTTTTCT--TATT--------TTGGCTTAGTTACGATTAATAGGAGTAGCTT-GGGGGCATTTG 420

PoC -----........T.......-......................................G.-.T...---............--....--------...............................-..A........ 420

pSH3 -----A..G.......T....-A....................T..T..C...TG..C.G.C-GTA..---T...G...C.T.GT....--------...................C....A...-..-........... 420

pDL4 -----A..G.......T....-A....................T..T..C...TG..C.G.C-GTA..---T...G...C.T.GT....--------...................C....A...-..-........... 420

pGZ1 -----A..G.......T....-A....................T..T..C...TG..C.G.C-GTA..---T...G...C.T.GT....--------...................C....A...-..-........... 420

pGZ2 -----A..G.......T....-A....................T..T..C...TG..C.G.C-GTA..---T...G...C.T.GT....--------...................C....A...-..-........... 420

pGZ3 -----A..G.......T....-A....................T..T..C...TG..C.G.C-GTA..---T...G...C.T.GT....--------...................C...........-........... 420

pHN1 -----................-........................................-.T...---....C.......--....--------...............................-........... 420

pHN3 -----A..G.......T....-A....................T..T..C...TG..C.G.C-GTA..---T...G...C.T.GT....--------...................C....A...-..-........... 420

pHN5 -----A..G.......T....-A....................T..T..C...TG..C.G.C-GTA..---T...G...C.T.GT....--------...................C....A...-..-........... 420

PvS ------GCA..--GC......T........................................-...A.---...TC.....T.TA.T.---------............................T..-....A...... 420

PvA ------GCA..--GC......-......................................G.-.T.T.---............TA.T.---------.-.............................-........... 420

PvO T--ATAGCA..--GC......C.............G..................G...A...-...TG---....A.......TA.T.---------.-..................T........A.-........... 420

PfA ------GCA.......T....-........T..........................CT..--.A.T.TTT............TA.T----------...............................-....A....C. 420

PfS ATGATTG.G..C.T..T....-.........................TA....T...CT..TT.T.T.TTTT...A..A..T.TT.TGATATTCTTA..A............................T.........C. 420

Pm ------ACAG..T...T....-......................................G.-.T.T.---............TA.T----------...............................-........... 420

Pk ------GCA...-G.......-......................................G.-.T.T.TTT............TA.T.---------.-.............................-........... 420

PoW GATTCAGATGTCAGAGGTGAAATTCTTAGATTTTCTGGAGACAAACAACTGCGAAAGCATTTGCCTAAAATACTTCCATTAATCAAGAACGAAAGTTAAGGGAGTGAAGACGATCAGATACCGTCGTAATCTTAACCATA 560

PoC T........................................................................................................................................... 560

pSH3 T..........T..............A.....A.........G.....T...........C..T....C...T..T................................................................ 560

pDL4 T.........................A.....A.........G.....T...........C..T....C...T..T................................................................ 560

pGZ1 T..........T..............A.....A.........G.....T...........C..T....C...T..T................................................................ 560

pGZ2 T..........T..............A.....A.........G.....T...........C..T....C...T..T................................................................ 560

pGZ3 T........................................................................................................................................... 560

pHN1 T........................................................................................................................................... 560

pHN3 T..........T..............A.....A.........G.....T...........C..T....C...T..T................................................................ 560

pHN5 T..........T..............A.....A.........G.....T...........C..T....C...T..T................................................................ 560

PvS T.........................A............................G.......T............................................A............................... 560

PvA T........................................................................................................................................... 560

PvO T.................................................................................................................T....G.................... 560

PfA T.........................................G....................T............................................................................ 560

PfSfS T.........................A...............GG..T.....................TC..T................................................................... 560

Pm T...........................................G............................................................................................... 560

Pk T........................................................................................................................................... 560

R6

R5

R5

R4

PoW AACTATGCCGACTAG-----GTTTTGGATGAAAGATTTTTAAATAAGAAAATTC-----CTTTTGG--AAATTTC-TTAG-----AT---TGCTTCCTTCAGTACCTTATGAGAAATCAAAGTCTTTGGGTTCTGGGGCG 700

PoC ...............-----..................................-----....C..GG.......-....-----..---.................................................. 700

pSH3 .........A....T-----..........T.T..GAAAA.GG.GGA.T.C..T-----G....ACGAC.T.G..A..CC-----..TTA......A............-.............................. 700

pDL4 .........A....T-----..........T.T..GAAAA.G..GGA.T.C..T-----G....ACGAC.T.G..A..CC-----..TTA......A............-.............................. 700

pGZ1 .........A....T-----..........T.T..GAAAA.GG.GGA.T.C..T-----G....ACGAC.T.G..A..CC-----..TTA......A............-.............................. 700

pGZ2 .........A....T-----..........T.T..GAAAA.GG.GGA.T.C..T-----G....ACGAC.T.G..A..CC-----..TTA......A............-.............................. 700

pGZ3 ...............-----..................................-----.......AA.TT.C.TAGAT-----------.................................................. 700

pHN1 .........A....T-----..........T.T..GAAAA.GG.GGA.T.C..T-----G....ACGAC.T.G..A..CC-----..TTA......A............-.............................. 700

pHN3 .........A....T-----..........T.T..GAAAA.GG.GGA.T.C..T-----G....ACGAC.T.G..A..CC-----..TTA......A............-.............................. 700

pHN5 .........A....T-----..........T.T..GAAAA.GG.GGA.T.C..T-----G....ACGAC.T.G..A..CC-----..TTA......A............-.............................. 700

PvS ......A........-----..............T.AAAC.......G.T.G.------..C..C.GGG.T---AG.CCT----T.GATT-T..............C................................. 700

PvA ...............-----.C.................A.........TT..------..C..C.GAGTT--.AT-.CT----T.GATT-............G.................................... 700

PvO ...A...........ACTAG.C...A.......AG....A........GTT..------..C..C.GAGTT--AACC.CT----T.GATT.............G.....G.G.........AC...............AA 700

PfA ...............-----..G...........TG..AA......A.GT------CAT....C.AGG-----.GAC.TT----T.GA-T.................................................. 700

PfS ......A........-----..G.........TATAAAAA.T...TA..T..GTAGCATT.C..A.GG..TG..GA..TTATATT.GAAT.................................................. 700

Pm ...............-----..G........T...G.AAA......A.G.GACA-----T.CA.ATAT.TGAG.GT..CTTT--T.GA-TA................................................. 700

Pk ...............-----.C.................A........GTT..TCTTTT..C.CC.GAG.T.AGAAC.CT----T.GATT-............G.................................... 700

PoW AGTATTCGCGCAAGCGAGAAAGTTAAAA-GAATTGACGGAAGGGCACCACCAGGCGTGGAGCTTGCGGCTTAATTTGACTCAACACGGGGAAACTCACTAGTTTAAGACAAGAGTAGGATTGACAGATTAATAG----CT 840

PoC ............................-......................................-..................................................................----.. 840

pSH3 ............................-............................................................A.....T......A...............................----.. 840

pDL4 ............................-........................A...................................A............A...............................----.. 840

pGZ1 ............................-............................................................A.....T......A...............................----.. 840

pGZ2 ............................-............................................................A.....T......A...............................----.. 840

pGZ3 ............................-.........................................................................................................----.. 840

pHN1 ............................-............................................................A.....T......A...............................----.. 840

pHN3 ............................-............................................................A.....T......A...............................----.. 840

pHN5 ............................-............................................................A............................................----.. 840

PvS ............................-............................................................A.......................................G.G..----.. 840

PvA ............................-............................................................A............................................----.. 840

PvO ............................A........................A............................G......A..G..............................A.....G.G..AGAG.. 840

PfA ............................-.........................................................................................................----.. 840

PfS ............................-............................................................A............................................----.. 840

Pm ............................-.........................................................................................................----.. 840

R7

R6

Pk ............................-............................................................A............................................----.. 840

PoW CTTTCTTGATTTCTTGGATGGTGATGCATGGCCGTTTTTAGTTCGTGAATATGATTTGTCTGGTTAATTCCGATAACGAACGAGATCTTAACCTGCTAATTAGCGGCGAATACGTTATATTCCTACTTGAAATT-GAATA 980

PoC ..........................................................................................................................T..TG.......-..... 980

pSH3 .....................................................................................C............G........CGG..T..CGCGAATT..-.CA....C-TT..C 980

pDL4 .....................................................................................C............G........CGG..T..CGCGAATT..-.CA....C-TT..C 980

pGZ1 .....................................................................................C............G........CGG..T..CGCGAATT..-.CA....C-TT..C 980

pGZ2 ......................................................................................................................................-..... 980

pGZ3 ......................................................................................................................................-..... 980

pHN1 .....................................................................................C............G........CGG..T..CGCGAATT..-.CA....C-TT..C 980

pHN3 .....................................................................................C............G........CGG..T..CGCGAATT..-.CA....C-TT..C 980

pHN5 ......................................................................................................................................-..... 980

PvS ..........................................................................................................TA.G....AC.....TT..TG.CGG...-.G..C 980

PvA ...........................................................................................................A......A.......T...G..GG.C.-....T 980

PvO ........................C.............C...........---.......G.......C.....G.T........C.............G.......A......A..C....T..GG.A.G...-...CC 980

PfA .............................................................................................................G...AC.......T..T........-...C. 980

PfS ..........................................................................................................TA.G...AC......TT..T........-..... 980

Pm ..........................................................................................................TA.....AC.......T..AG.......A..... 980

Pk ...........................................................................................................A......A.......T..TG.AG....-..... 980

PoW TAGCTGAATTT-GCTTATTTTGAAGAATAT------------ATTAGGAT-AC--A-TTA--TAGTGTCCTTTTCCC---------------------------------TTTTCTACTTAATTCGCTAT---------- 1120

PoC ...........-T................C------------........-..--.A...--AT.............---------------------------------.A...............A..---------- 1120

pSH3 .C.-.AGGC.GGAT.AT....AG.AG....------------GACG.ATA-..CG.G.CGAG.GTC....CCA..A.---------------------------------.G.....-.........AG.---------- 1120

pDL4 .C.-.AGGC.GGAT.AT....AG.AG....------------GACG.ATA-..CG.G.CGAG.GTC....CCA..A.---------------------------------.G.....-.........AG.---------- 1120

pGZ1 .C.-.AGGC.GGAT.AT....AG.AG....------------GACG.ATA-..CG.G.CGAG.GTC....CCA..A.---------------------------------.G.....-.........AG.---------- 1120

pGZ2 ...........-..................------------........-G.--.-...--...............---------------------------------..............T.....---------- 1120

pGZ3 ...........-..................------------........-G.---A...--...............---------------------------------..............T.....---------- 1120

pHN1 .C.-.AGGC.GGAT.AT....AG.AG....------------GACG.ATA-..CG.G.CGAG.GTC....CCA..A.---------------------------------.G.....-.........AG.---------- 1120

pHN3 .C.-.AGGC.GGAT.AT....AG.AG....------------GACG.ATA-..CG.G.CGAG.GTC....CCA..A.---------------------------------.G.....-.........AG.---------- 1120

pHN5 ...........T.................C------------........-..--.A...--...............---------------------------------..............T.....---------- 1120

PvS .G.A...T.-.GCT.-..A....G.TGC.ATCTAAATAGGGG...GCA..T.TACTTCGTGTCG....TTC..AAT.GAATAGCTGATGCGTTTGGTATATTGCTTTCCT....T.TA..TC.G....TCTTTACTTGGC 1120

PvA CG.T...T.-.GCT.-.C..C......A..------------...G....------ACGTAAC...T...C......---------------------------------...........G......T.---------- 1120

PvO .G.T...T.-.GCTC-....C....TGC.CGCATGA----GG..CG....T.TGTGACGCGTC...T.TT..C.G..GATTTACTGATGCAGCACATAT-----TTCTCG..C.T.CA..CG.G.A..TCGAAA-TAAGC 1120

PfA ...G.A.C.A.ACA.--..A.TC..T.ATC----------AA........-.TTTTT---AT..AAA.ATCC..TT.-----------------------------CCTG..C.ACTAA...A.T.T.T.TT-------- 1120

PfS ...G.A.T.A.ACA.GT..A.TC..TG.TC----------AA........-.TTTTT..TAT..AAA.AT.C..TT.-----------------------------CCTG....ACTAA.....T.T.T.TTT------- 1120

Pm ...A.A...-.GTGC..A...TG.TT.A.AT-----------.....A..GTTTTTT...ATA.AAACGT.C..TT.----------------------------CCTTT......TAA.T..G.ATAT.---------- 1120

Pk ...TG..T.-.GTTGG...........A..------------...G.A..T..GTTAAATGTG.T.CCTT.CCC------------------------------------..............TA.AT.---------- 1120

R8

R7

PoW ---TCATGCTGTTTCTTT-TTTGTGTAGGAATGT-----ATTCGTTTGATTGTA--AAGCTTCTTAGAGGAACGATGTGTGTCTAACACAAGGAAGTTTAAGGCAACAACAGG 1233

PoC ---.............C.-....CA.........-----...............--............................................T............ 1233

pSH3 ---CTG.C...G..T...-...TG...A.T....-----..C..C....A....--.-............T..........CT.............................. 1233

pDL4 ---CTG.C...G..T...-...AG...A.T....-----..C..C....A....--.-............T..........CT.............................. 1233

pGZ1 ---CTG.C...G..T...-...-G...A.T....-----..C..C....A....--.-............T..........CT.............................. 1233

pGZ2 ---...............-...............-----...............--......................................................... 1233

pGZ3 ---...............-...............-----...............--......................................................... 1233

pHN1 ---CTG.C...G..T...-...-G...A.T....-----..C..C....A....--.-............T..........CT.............................. 1233

pHN3 ---CTG.C...G..T...-...-G...A.T....-----..C..C....A....--.-............T..........CT.............................. 1233

pHN5 ---...............-...............-----...............--......................................................... 1233

PvS TTA..G.A.C.....C..-.........A.....-----...T.CA.T..AT..--......................................................... 1233

PvA ---....A..........-..C.C...A......-----...T.C.........--.........................C............................... 1233

PvO AAA...AC.A.G...AA.-...ACC..A......GTTTC...T.C.........--.................AG.......G..........................A.CA 1233

PfA -----.CT..A.....C.C..CT.T..A......-----.C.T.C.......A.--.................AT...................................... 1233

PfS ---.T.CT..A.....C.C..CT.T..A......-----.C.T.C......AA.TA.................AG.....A................................ 1233

Pm ----T..T..T.......T..C.CA..A......-----...T.C..A......--......................................................... 1233

Pk ---..CAT..A.......-....C...T......-----...T.C.........--....................................--------------------- 1233
